# Interactive 3D segmentation for primary gross tumor volume in oropharyngeal cancer

**DOI:** 10.1038/s41598-025-13601-3

**Published:** 2025-08-05

**Authors:** Mikko Saukkoriipi, Jaakko Sahlsten, Joel Jaskari, Lotta Orsmaa, Jari Kangas, Nastaran Rasouli, Roope Raisamo, Jussi Hirvonen, Helena Mehtonen, Jorma Järnstedt, Antti Mäkitie, Mohamed Naser, Clifton Fuller, Benjamin Kann, Kimmo Kaski

**Affiliations:** 1 Department of Computer Science, Aalto University School of Science, Espoo, Finland; 2 Faculty of Information Technology and Communication Sciences, Computing Sciences, University of Tampere, Tampere, Finland; 3 Department of Radiology, Tampere University, Faculty of Medicine and Health Technology, Tampere, Finland; 4 Department of Otorhinolaryngology–Head and Neck Surgery, Research Program in Systems Oncology, Faculty of Medicine, University of Helsinki and Helsinki University Hospital, Helsinki, Finland; 5 Department of Radiation Oncology, The University of Texas MD Anderson Cancer Center, Houston, TX USA; 6 Artificial Intelligence in Medicine Program, Mass General Brigham, Harvard Medical School, Boston, MA USA; 7 Department of Radiation Oncology, Dana-Farber Cancer Institute and Brigham and Women’s Hospital, Harvard Medical School, Boston, MA USA; 8 The Alan Turing Institute, British Library, 96 Euston Rd, NW1 2DB London, United Kingdom

**Keywords:** Cancer, Image processing

## Abstract

Radiotherapy is the main treatment modality of oropharyngeal cancer (OPC), in which an accurate segmentation of primary gross tumor volume (GTVt) is essential but also challenging due to significant interobserver variability and the time consumed in manual tumor delineation. For such a challenge an interactive deep learning (DL) based approach offers the advantage of automatic high-performance segmentation with the flexibility for user correction when necessary. In this study, we investigate an interactive DL for GTVt segmentation in OPC by introducing a novel two-stage Interactive Click Refinement (2S-ICR) framework and implementing state-of-the-art algorithms. Using the 2021 HEad and neCK TumOR dataset for development and an external dataset from The University of Texas MD Anderson Cancer Center for evaluation, the 2S-ICR framework achieves a Dice similarity coefficient of 0.722 ± 0.142 without user interaction and 0.858 ± 0.050 after ten interactions, thus outperforming existing methods in both cases.

## Introduction

Oropharyngeal cancer (OPC) is a subtype of head and neck squamous cell carcinoma that predominantly affects the tonsils and the base of the tongue and poses substantial challenges in medical imaging and treatment. Early detection and effective treatment of OPC are critical for improving patient outcomes, in terms of quality of life and survival^1^. Magnetic Resonance Imaging (MRI), Computed Tomography (CT), and Positron Emission Tomography (PET) are the primary modalities used for the initial staging, planning of radiation therapy (RT), and follow-up of OPC^2^. RT is a pivotal treatment modality for OPC, but it is based on laborious and error-prone manual or semi-automatic segmentation of primary gross tumor volume (GTVt)^2^. An accurate segmentation of GTVt in the oropharynx region is particularly challenging due to significant interobserver variability^3–5^. This challenge not only compromises the efficacy of treatment, but it also increases both the duration and cost of care^2^. Consequently, there is a need for the development of precise, fast, and cost-efficient automatic segmentation techniques for OPC GTVt to enhance treatment outcomes and operational efficiency.

An automated segmentation of the OPC GTVt using deep learning (DL) methods has shown good promise in reducing variability and enhancing the precision and reliability of radiotherapy planning^6–8^. However, the segmentation can fall short of the required performance and necessitate further manual refinement or complete rework by clinicians. In such cases, interactive deep learning presents a compelling approach by facilitating efficient interface for segmentation refinement^9^.

A common approach for interactive DL segmentation is click-based interaction, where the user provides feedback by clicking on coordinates requiring correction^9,10^. To our knowledge, the only work considering interactive DL for OPC tumor segmentation is^11^, which used a slice-based method where users manually segment an entire slice of the tumor volume. However, this approach requires time-consuming retraining of the DL model after each interaction, limiting its practical use in clinical settings. Consequently, research on practical interactive deep learning segmentation to improve GTVt segmentation remains limited, despite its potential benefits^9^.

As for related research works, the DeepGrow^12^ and DeepEdit^13^, both integrated within the Medical Open Network for Artificial Intelligence (MONAI)^14^, are two widely known click-based interactive segmentation methods. The DeepGrow incorporates interaction events in each iteration during training, which leads to lower initial segmentation performance compared to the baseline. However, with interactions, segmentation accuracy improves rapidly^12,13^. The DeepEdit addresses this issue by introducing a hyperparameter called “click-free iterations,” which controls the fraction of non-interactive training iterations. While this improves initial segmentation, it affects negatively the performance during interactions^13^. Evaluations of multiple 3-dimensional (3D) medical image data sets have shown that DeepEdit performance is inferior to traditional DL segmentation methods in non-interactive mode, and inferior to DeepGrow in interactive mode^13^. Hence for both methods there is trade-off between non-interactive and interactive segmentation performance.

In this study, we introduce a novel two-stage Interactive Click Refinement (2S-ICR) framework to enhance user-driven segmentation refinement while preserving the initial state-of-the-art segmentation accuracy. In addition we demonstrate the effectiveness of interactive OPC GTVt segmentation from volumetric PET-CT scans and highlight its potential in clinical applications. Moreover, we conduct a comprehensive comparison of existing click-based state-of-the-art interactive deep learning methods against our proposed segmentation framework, which establishes a new benchmark for future research on OPC GTVt segmentation.

Our findings reveal that the trade-off between the non-interactive and interactive segmentation performance can be addressed by dividing the task into two stages and training specialized deep learning models for each stage. We refer to these models as the initial and refinement networks. Additionally, we find that the sigmoid probability volume can be used efficiently as a memory mechanism, not only between the non-interactive and interactive deep learning models but also across interaction events. Furthermore, we demonstrate that an ensemble approach can be seamlessly integrated into interactive semantic segmentation.

## Results

### Experimental setup

We trained and validated interactive deep learning models using five-fold cross-validation on the 2021 HECKTOR dataset^2^ and simulated user interactions as in^12,13^. To reduce variability due to simulated probabilistic interactions, validation was repeated three times with different seeds. Testing on the MDA dataset employed an ensemble of the five-fold trained HECKTOR 2021 models. DeepEdit was trained with 0%, 25%, and 50% click-free propotions, referred to as DeepGrow, DeepEdit-25, and DeepEdit-50, respectively.

### Segmentation performance

Initial segmentation, or 0-click segmentation, represents the model output before any user interactions. The 2S-ICR framework demonstrated superior Dice Similarity Coefficient performance for the HECKTOR 2021 and MDA datasets. On the MDA dataset, 2S-ICR achieved a DSC of 0.722, surpassing DeepGrow with 0.642 and DeepEdit variants with scores of 0.642 and 0.721 for 25 percent and 50 percent click-free propotions. On the HECKTOR 2021 dataset, 2S-ICR achieved the highest DSC of 0.752, exceeding DeepGrow with 0.663 and DeepEdit models with scores of 0.729 and 0.738. For the HD95, the 2S-ICR achieved the best result on the HECKTOR 2021 dataset with a value of 3.000. On the MDA dataset, the 2S-ICR achieved a value of 5.385, just next to DeepEdit-50 with value of 5.099. Full results for HECKTOR 2021 and MDA datasets are shown in Tables 1 and 2, respectively.

**Table 1:** Quantitative results on the MDA dataset ($N = 67$) for 0, 1, 5, and 10 number of clicks and for the overall averaged. Bolded values indicate the best performance in each column.

Model	0 Clicks	1 Click	5 Clicks	10 Clicks	0 to 10 Avg.
(a) Dice similarity coefficient performance					
2S-ICR (ours)	**0.722** ± **0.142**	**0.773** ± **0.128**	**0.835** ± **0.072**	**0.858** ± **0.050**	**0.820** ± **0.097**
DeepGrow^12,13^	0.642 ± 0.216	0.722 ± 0.139	0.818 ± 0.074	0.849 ± 0.051	0.794 ± 0.119
DeepEdit-25^13^	0.642 ± 0.151	0.738 ± 0.141	0.806 ± 0.081	0.839 ± 0.055	0.795 ± 0.104
DeepEdit-50^13^	0.721 ± 0.153	0.739 ± 0.145	0.791 ± 0.093	0.822 ± 0.074	0.783 ± 0.112
(b) HD95 performance					
2S-ICR (ours)	5.385 ± 5.124	**4.472** ± **3.325**	**3.000** ± **1.506**	**2.236** ± **0.764**	**3.000** ± **2.065**
DeepGrow^12,13^	6.403 ± 10.368	5.099 ± 4.457	3.317 ± 1.909	2.449 ± 0.926	3.317 ± 2.551
DeepEdit-25^13^	5.385 ± 5.144	4.690 ± 3.620	3.317 ± 2.133	2.828 ± 1.506	3.606 ± 2.650
DeepEdit-50^13^	**5.099 ± 6.636**	4.899 ± 4.211	3.606 ± 2.551	3.000 ± 1.887	3.606 ± 2.650

(a) Dice similarity coefficient (mean ± standard deviation) performance. (b) HD95 (median ± interquartile range) performance in millimeters.

**Table 2:** Quantitative results on the HECKTOR 2021 dataset over 5-fold validation for 0, 1, 5, and 10 number of clicks and for the overall averaged.

Model	0 Clicks	1 Click	5 Clicks	10 Clicks	0 to 10 Avg.
(a) Dice similarity coefficient performance					
2S-ICR (ours)	**0.752** ± **0.203**	**0.787** ± **0.191**	**0.850** ± **0.101**	**0.870** ± **0.067**	**0.836** ± **0.131**
DeepGrow^12,13^	0.663 ± 0.316	0.755 ± 0.211	0.840 ± 0.096	0.861 ± 0.065	0.817 ± 0.157
DeepEdit-25^13^	0.729 ± 0.243	0.767 ± 0.197	0.828 ± 0.110	0.851 ± 0.076	0.814 ± 0.144
DeepEdit-50^13^	0.738 ± 0.229	0.765 ± 0.200	0.822 ± 0.128	0.847 ± 0.094	0.812 ± 0.150
(b) HD95 performance					
2S-ICR (ours)	**3.000 **± **4.103**	**2.236** ± **2.123**	**2.000** ± **1.414**	**1.732** ± **0.822**	**2.000** ± **1.586**
DeepGrow^12,13^	3.674 ± 9.273	3.000 ± 3.099	**2.000 ± 1.268**	2.000 ± 1.035	2.236 ± 1.430
DeepEdit-25^13^	3.317 ± 6.690	3.000 ± 3.385	2.236 ± 1.430	2.000 ± 1.414	2.236 ± 1.874
DeepEdit-50^13^	3.081 ± 5.413	3.000 ± 3.385	2.236 ± 1.430	2.000 ± 1.414	2.236 ± 1.585

Each interaction and sample outcome is averaged over three repetitions, and the dataset (N=224) mean and standard deviation are reported. Bolded values indicate the best performance in each column. (a) Dice similarity coefficient (mean ± standard deviation) performance. (b) HD95 (median ± interquartile range) performance in millimeters.

Specifically on the MDA dataset, the 2S-ICR showed steady improvements for all interaction levels. Specifically, the DSC increased from 0.722 for 0 clicks to 0.858 for 10 clicks, with an average performance of 0.820 over the whole range of interaction. In terms of HD95, the metric improved significantly from 5.385 mm for 0 clicks to 2.236 mm for 10 clicks, thus indicating enhanced boundary precision through user interactions.

Specifically on the HECKTOR 2021 dataset, the 2S-ICR achieved the highest DSC of 0.836 on average, with a peak of 0.870 for 10 clicks. The HD95 results paralleled the improvements in the DSC, starting from 3.000 mm and down to 1.732 mm by the tenth interaction. These results reflect a consistent enhancement in segmentation accuracy with increasing user input.

**Fig. 1:**
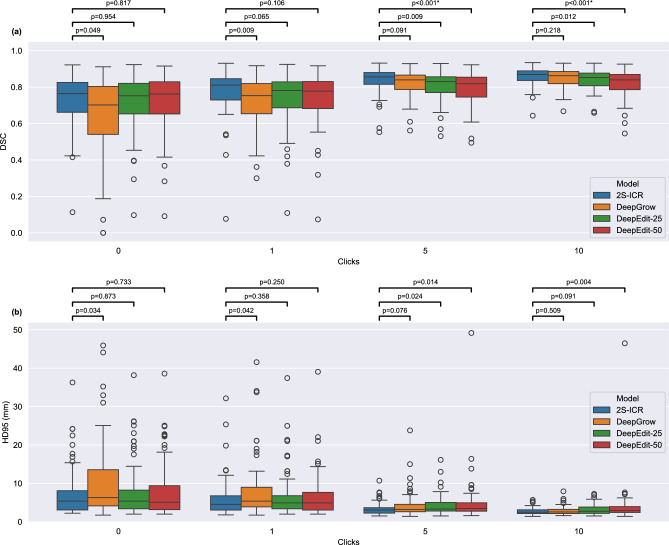
Change in the segmentation performance through click interactions evaluated on the MDA dataset. Performance is evaluated using (**a**) Dice similarity coefficient (DSC) and (**b**) Hausdorff Distance at the 95th percentile (HD95). The statistical significance tests between the models are based on the two-sided Wilcoxon signed rank test with Benjamini–Hochberg procedure to correct for multiple testing, in which p < 0.05 is considered significant.

Compared to DeepGrow and DeepEdit variants, the 2S-ICR consistently delivered the best results. On the MDA dataset, it maintained the highest DSC of 0.820 on average, compared to DeepGrow’s 0.794 and DeepEdit-25’s 0.795. HD95 results further emphasized its superiority, showing the most substantial improvements across all interaction levels. Statistical analysis in Fig. 1 revealed that the 2S-ICR is statistically significantly better than DeepEdit-25 and DeepEdit-50 after 5 and 10 clicks.

### Number of clicks required to achieve specific thresholds

The metric of number of clicks (NoC) highlights the efficiency of the 2S-ICR framework in achieving specific segmentation thresholds. On the MDA dataset, the 2S-ICR consistently required fewer clicks compared to the DeepGrow and DeepEdit variants. For example, it achieved a Dice Similarity Coefficient (DSC) of 0.75 with just 1.81 clicks on average, and a DSC of 0.85 with 5.97 clicks. Additionally, 2S-ICR showed superior performance for HD95 thresholds, requiring fewer interactions to achieve 5.0 mm and 2.5 mm thresholds.

Similarly, on the HECKTOR 2021 dataset, the 2S-ICR mostly outperformed the competing methods for all metrics, see Table 3. It achieved the DSC value of 0.75 with an average of 1.34 clicks and 0.85 with 3.96 clicks. For the HD95 threshold of 5.0 mm, 2S-ICR required only 0.07 clicks on average. Moreover, the proportion of failures (PoF) was the lowest for 2S-ICR in nearly all cases, except for the 0.75 DSC threshold on HECKTOR 2021, where the DeepGrow showed a marginally lower failure rate of 0.45% compared to the 2S-ICR’s rate of 1.64%.

**Table 3:** Number of Clicks (NoC) required to achieve specific thresholds for Dice Similarity Coefficient (DSC) and Hausdorff Distance at the 95th percentile (HD95) on the MDA and HECKTOR 2021 datasets.

Method	0.75 DSC		0.85 DSC		5.0 mm HD95		2.5 mm HD95	
	NoC (cnt)	PoF (%)	NoC (cnt)	PoF (%)	NoC (cnt)	PoF (%)	NoC (cnt)	PoF (%)
(a) Number of Clicks (NoC) required to achieve specific thresholds on the MDA dataset ($N = 67$), using an ensemble of models trained via 5-fold cross-validation on the HECKTOR 2021 dataset.								
2S-ICR (ours)	$\mathbf {1.81 \pm 0.12}$	$\mathbf {0.00 \pm 0.00}$	$\mathbf {5.97 \pm 0.25}$	$\mathbf {15.92 \pm 1.41}$	$\mathbf {1.00 \pm 0.00}$	$\mathbf {0.00 \pm 0.00}$	$\mathbf {6.50 \pm 0.41}$	$\mathbf {17.41 \pm 1.86}$
DeepGrow^12,13^	$2.50 \pm 0.06$	$1.49 \pm 0.00$	$7.40 \pm 0.29$	$20.90 \pm 2.11$	$2.00 \pm 0.00$	$0.00 \pm 0.00$	$8.00 \pm 0.00$	$21.89 \pm 1.41$
DeepEdit-25^13^	$2.67 \pm 0.12$	$1.00 \pm 0.70$	$7.50 \pm 0.08$	$26.37 \pm 0.70$	$1.33 \pm 0.47$	$0.00 \pm 0.00$	$8.00 \pm 0.41$	$28.86 \pm 1.86$
DeepEdit-50^13^	$3.46 \pm 0.09$	$2.49 \pm 0.70$	$8.46 \pm 0.56$	$15.92 \pm 1.41$	$1.00 \pm 0.00$	$3.48 \pm 0.70$	$10.17 \pm 0.62$	$34.83 \pm 0.70$
(b) Number of Clicks (NoC) required to achieve specific thresholds on the HECKTOR 2021 dataset ($N = 224$), trained using 5-fold cross-validation.								
2S-ICR (ours)	$\mathbf {1.34 \pm 0.46}$	$1.64 \pm 1.72$	$\mathbf {3.96 \pm 0.97}$	$\mathbf {16.38 \pm 5.45}$	$0.07 \pm 0.25$	$\mathbf {0.30 \pm 0.77}$	$\mathbf {0.97 \pm 0.64}$	$\mathbf {6.27 \pm 3.11}$
DeepGrow^12,13^	$2.05 \pm 0.41$	$\mathbf {0.45 \pm 0.90}$	$5.02 \pm 0.51$	$19.52 \pm 4.17$	$0.40 \pm 0.49$	$0.30 \pm 0.76$	$1.90 \pm 1.17$	$8.80 \pm 4.42$
DeepEdit-25^13^	$2.41 \pm 1.16$	$1.05 \pm 1.12$	$4.97 \pm 1.13$	$25.13 \pm 13.32$	$0.33 \pm 0.47$	$0.45 \pm 0.91$	$4.20 \pm 3.86$	$10.01 \pm 5.90$
DeepEdit-50^13^	$2.44 \pm 0.65$	$1.20 \pm 1.80$	$4.54 \pm 0.91$	$25.00 \pm 7.08$	$\mathbf {0.00 \pm 0.00}$	$1.49 \pm 1.78$	$2.70 \pm 1.93$	$11.33 \pm 4.90$

Values are reported as mean ± standard deviation. PoF (%) indicates the proportion of images that failed to reach the given threshold within 20 interaction events. Bolded values indicate the best performance in each category.

**Fig. 2:**
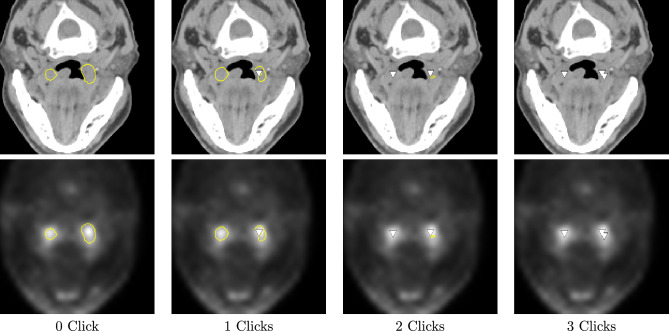
The progressive refinement of segmentation of 2S-ICR shown for the first three interactions overlaid on CT (top row) and PET (bottom row) slices. In addition, false positives are marked in yellow and clicks with white arrow.

Segmentation refinement using the 2S-ICR framework is visually demonstrated with a scan from the MDA test set in the Fig. 2. Initially, the network segmented two regions adjacent to the throat erroneously, as highlighted in yellow. These regions were connected to a tumor located in the lower horizontal region of the neck, resulting in an overly extensive segmentation mask. Through user interactions, the segmentation surface was iteratively adjusted to align more closely with the ground truth delineation. Specifically, the segmentation on the left side was refined with a single click, while the right side required two additional clicks, for optimal correction. This example illustrates the capability of the method to efficiently enhance segmentation accuracy by guiding the segmentation surface closer to the ground truth tumor boundaries through user interaction, as depicted in Fig. 2.

### Runtime and memory analysis

**Table 4:** Inference efficiency of interactive segmentation methods on the HECKTOR 2021 dataset, reporting peak VRAM usage and mean inference times (with standard deviation) on an NVIDIA RTX 3080 GPU and Intel i5-12600K CPU.

Method	VRAM (GB)	NVIDIA RTX 3080 (10 GB)	Intel i5-12600K CPU
		Inference time (s)	Inference time (s)
2S-ICR (ours)	2.06	$0.08\pm 0.00$	$1.62\pm 0.05$
DeepGrow^12,13^	1.86	$0.08\pm 0.01$	$1.63\pm 0.05$
DeepEdit-25^13^	1.86	$0.08\pm 0.01$	$1.63\pm 0.05$
DeepEdit-50^13^	1.86	$0.08\pm 0.01$	$1.63\pm 0.05$
U-Net^15^	1.88	$0.08\pm 0.00$	$1.63\pm 0.05$

U-Net^15^ is the non-interactive baseline.

We evaluate the inference efficiency of our proposed 2S-ICR method against baseline interactive segmentation approaches–DeepGrow^12^, DeepEdit variants (DeepEdit-25, DeepEdit-50)^13^, and a non-interactive U-Net baseline^15^–on 3D volumes from the HECKTOR 2021 dataset. Tests were conducted on an NVIDIA RTX 3080 GPU (10 GB VRAM) and an Intel i5-12600K CPU, with results summarized in Table 4.

As shown in Table 4, 2S-ICR achieves a GPU inference time of 0.08 s, matching the efficiency of DeepGrow, DeepEdit variants, and U-Net, with a peak VRAM usage of 2.06 GB. This VRAM requirement, while slightly higher than DeepGrow/DeepEdit (1.86 GB) and U-Net (1.88 GB), remains well within the capacity of consumer-grade GPUs to ensure practical deployment. On the CPU, 2S-ICR delivers a competitive inference time of 1.62 ± 0.05 s, marginally faster than the 1.63 ± 0.05 s of baselines. These results demonstrate that 2S-ICR balances low latency and modest memory demands, making it well-suited for real-time interactive segmentation in clinical workflows.

### Impact of mask dropout on interactive segmentation performance

The incorporation of mask dropout during training of the 2S-ICR framework influenced significantly interactive segmentation performance. As presented in Table 5, the DSC increased from $0.827 \pm 0.134$ to $0.845 \pm 0.109$ when $p_{\text {drop}}$ increased from 0 to 0.2. For higher dropout probabilities, the DSC values stabilized around 0.845, showing minimal sensitivity for further increases in $p_{\text {drop}}$.

Increasing $p_{\text {drop}}$ from 0.0 to 0.2 had a significant impact on the number of voxels affected per interaction event. As can be seen in Table 5, for $p_{\text {drop}} = 0.0$, the mean number of voxels adjusted was $731 \pm 719$, beign significantly lower compared to the $941 \pm 1477$ for $p_{\text {drop}} = 0.2$. This increase suggests that introducing mask dropout facilitates larger updates in response to user interactions. Beyond $p_{\text {drop}} = 0.2$, the mean number of changed voxels varied only slightly, stabilized around $900$.

**Table 5:** Effect of varying mask dropout probability ($p_{\text {drop}}$) on the interactive segmentation performance of the 2S-ICR framework on the HECKTOR 2021 dataset.

$p_{\text {drop}}$	DSC 1 to 10 Avg.	N changed voxels
0.0	$0.827 \pm 0.134$	$731 \pm 719$
0.2	$0.845 \pm 0.109$	$941 \pm 1477$
0.4	$0.843 \pm 0.112$	$888 \pm 1075$
0.6	$0.843 \pm 0.116$	$932 \pm 1161$
0.8	$0.842 \pm 0.104$	$917 \pm 1208$

The table presents the average Dice similarity coefficient (DSC) and the mean number of voxels changed per interaction event ($\mu \pm \sigma$). Results highlight the impact of different $p_{\text {drop}}$ values on segmentation accuracy and model adaptability to user interactions.

## Discussion

Here we have introduced a two-stage click refinement (2S-ICR) framework, to serve as a novel interactive deep learning method that redefines the standard for segmenting the volume of primary gross tumors in oropharyngeal cancer. Our framework’s core innovation lies in the deployment of two specialized models: an initial segmentation model and a refinement model. This dual-model approach strategically eliminates the trade-off between non-interactive and interactive performance, as observed in case of previous methodologies.

Volumetric medical image segmentation offers significant potential but is fraught with unique challenges in the medical field. These include heterogeneous data from various imaging devices, imaging artifacts, patient-specific variations, disparate image acquisition and quality across centers, and the presence of lymph nodes with high metabolic responses in PET images^2^. These complexities can occasionally lead to failures in AI-driven segmentation, thus highlighting the indispensable need for human expertise to interactively guide and refine the segmentation process with AI models. Given the current limitations of technology, this collaboration between human expertise and AI models is essential to achieve precise and reliable results in medical imaging.

Although the interactive segmentation approach has a strong basis in 2D, particularly in non-medical domains^16–18^, the previous interactive segmentation research in the OPC GTVt domain has mainly focused on 2D slicing methods^11^ or on reducing annotation effort^19^. However, the state-of-the-art non-interactive segmentation methods for OPC GTVt have used 3D methods with volumetric PET-CT scans and were found to improve performance via global context compared to 2D based methods^2,6–8,20^. Our present work addresses this limitation by performing interactive OPC GTVt segmentation directly in the volumetric space.

Although some 2D interactive segmentation methods employ two models to reduce computational costs^21^, our motivation for the two-model architecture of 2S-ICR is distinct. Here we will prioritize avoiding the trade-off between non-interactive and interactive performance as is often the case in single-model approaches^13^. By leveraging previous outputs as input, a common practice in 2D shown to stabilize predictions^18^, we not only enhance 3D performance but also seamlessly chain non-interactive and interactive models. This enables a synergistic workflow, in which each model is optimized for its specific task.

DeepEdit was one of the first interactive models implemented for 3D medical segmentation tasks^13^, where both the pre- and post-interaction performances were measured. It turned out that the quality of interactive DL segmentation without interactions was worse than that of non-interactive DL methods. DeepEdit has addressed this issue, to some extent, with the approach of “click-free” (i.e., non-interactive) training iterations. However, this approach introduced a trade-off: more click-free iterations improved non-interactive performance at the expense of interactive performance. In contrast, the 2S-ICR’s two distinct models ensure optimal initial segmentation and effective refinement with interactions.

In the evaluation of our framework using the MDA dataset, 2S-ICR turned out to consistently outperform user interactions at all levels compared to established methods such as DeepGrow and various configurations of DeepEdit, with the only exception being HD95 at 0 clicks. However, as HD95 was not used during training, this also illustrates the discrepancy between DSC and HD95 results. Specifically, the Dice similarity coefficients for the 2S-ICR ranged from 0.722 without any clicks to 0.858 with ten clicks, averaging 0.820 across all interaction levels, which exceeds the performance of competing models (Table 1). These results underscore the efficacy of our dual-model architecture in harnessing user interactions to progressively refine segmentation accuracy without compromising baseline performance.

Moreover, the 2S-ICR showed superior handling of segmentation challenges, as evidenced by the HD95 results. For example, HD95 metrics improved from 5.385 mm at 0 clicks to 2.236 mm at 10 clicks, with a lower interquartile range than in case of other methods, reflecting a stable and substantial improvement in segmentation quality as user involvement increased (Table 1). These results not only highlight the robustness of 2S-ICR in different operational scenarios, but also shows its potential to deliver precise and clinically relevant segmentation in interactive settings.

The analysis of the HECKTOR and MDA datasets reveal a significant variance in the image-level segmentation results, which can be seen in Tables 1 and 2, respectively, confirming the challenges noted in the existing literature on accurate GTVt segmentation^2,3,5,20^. This variability shows that while some segmentations meet clinical standards, others do not, which emphasizes the need for an interactive method for efficiently enhancing suboptimal segmentations.

Beyond segmentation accuracy, the inference efficiency of 2S-ICR underscores its potential for clinical adoption. On an NVIDIA RTX 3080 GPU, 2S-ICR achieves an inference time of 0.08 s and matches the performance of established interactive methods like DeepGrow and DeepEdit, while using 2.06 GB of VRAM. Although this VRAM usage is slightly higher than baselines (1.86–1.88 GB), it remains well within the capacity of consumer-grade GPUs. On an Intel i5-12600K CPU, 2S-ICR delivers a low latency result of 1.62 ± 0.05 s and enables real-time segmentation on standard clinical workstations without specialized hardware. These attributes highlight 2S-ICR’s suitability for seamless integration into clinical workflows.

Our study has several limitations. First, the 2S-ICR framework was developed and evaluated for a single binary segmentation task, whereas clinical applications often require multi-class segmentation, such as distinguishing primary tumours from lymph nodes^20^. While 2S-ICR is theoretically extendable to multi-class interactive segmentation–for example, by incorporating class-specific positive and negative click maps (i.e., 3D volumes encoding user interactions for each class) and modifying the output layer accordingly–this extension is beyond the scope of the present study and thus left for future work.

Second, the evaluation was limited to primary gross tumour volumes using the HECKTOR 2021^2^ and MDA datasets, which, although derived from real-world clinical settings, do not include complex cases such as metal artifacts or post-surgical anatomy. As prior work has shown that evaluation outcomes are sensitive to dataset composition^22^, and therefore, the generalizability of our results to other clinical scenarios is uncertain. However, as for reducing the effects of metal artifacts, we refer the reader to the following literature^23,24^. In addition, 2S-ICR could potentially be integrated into active learning pipelines^25,26^ to support efficient annotation of prioritized samples.

Third, we used simulated interaction events, following prior work^12,13^. Although simulations provide a controlled and scalable environment, they may not fully capture how clinicians interact in practice. The simulator identifies error regions by comparing model predictions to ground truth and samples interaction points using a distance-weighted probability distribution. While effective for benchmarking, this approach assumes idealized user behavior by favoring areas with large errors and never producing incorrect interaction events. Furthermore, our preliminary results indicated that the location of the interaction had a considerable effect on the model performance. This highlights the need to understand clinician behavior during interactive segmentation for improved applicability. To develop better-suited interaction simulation algorithms, human interaction patterns should be analysed, and improved simulation algorithms should be developed. As interactive segmentation changes depend on interaction locations and types, these may affect the results. However, this study was beyond the scope and is planned for future work.

Despite these limitations, 2S-ICR remains a flexible framework that may generalize to broader clinical applications, support active learning, and be adapted to various segmentation architectures beyond the one evaluated in this study.

The benefits of the proposed framework extend beyond improved accuracy. By eliminating the last remaining drawback associated with interactive segmentation, 2S-ICR unlocks the full potential of the entire interactive segmentation field. This breakthrough paves the way for a wider adoption of interactive segmentation in various clinical applications. By enabling clinicians to easily and quickly improve segmentation results, it promises more accurate treatment planning and improved patient outcomes.

In this study we have introduced 2S-ICR, a new interactive click-based framework, for segmentation of primary gross tumor volume in oropharyngeal cancer. The results show that our framework achieves performance comparable to or superior to state-of-the-art interactive deep learning methods, both with and without user interactions. These results highlight the potential of this approach to improve the performance of GTVt segmentation, enabling clinicians to quickly improve segmentation results based on just a few interactions. The more accurate segmentation enabled by our approach could lead to a more precise OPC treatment planning and to improved patient outcomes.

## Methods

### 2S-ICR framework

**Fig. 3:**
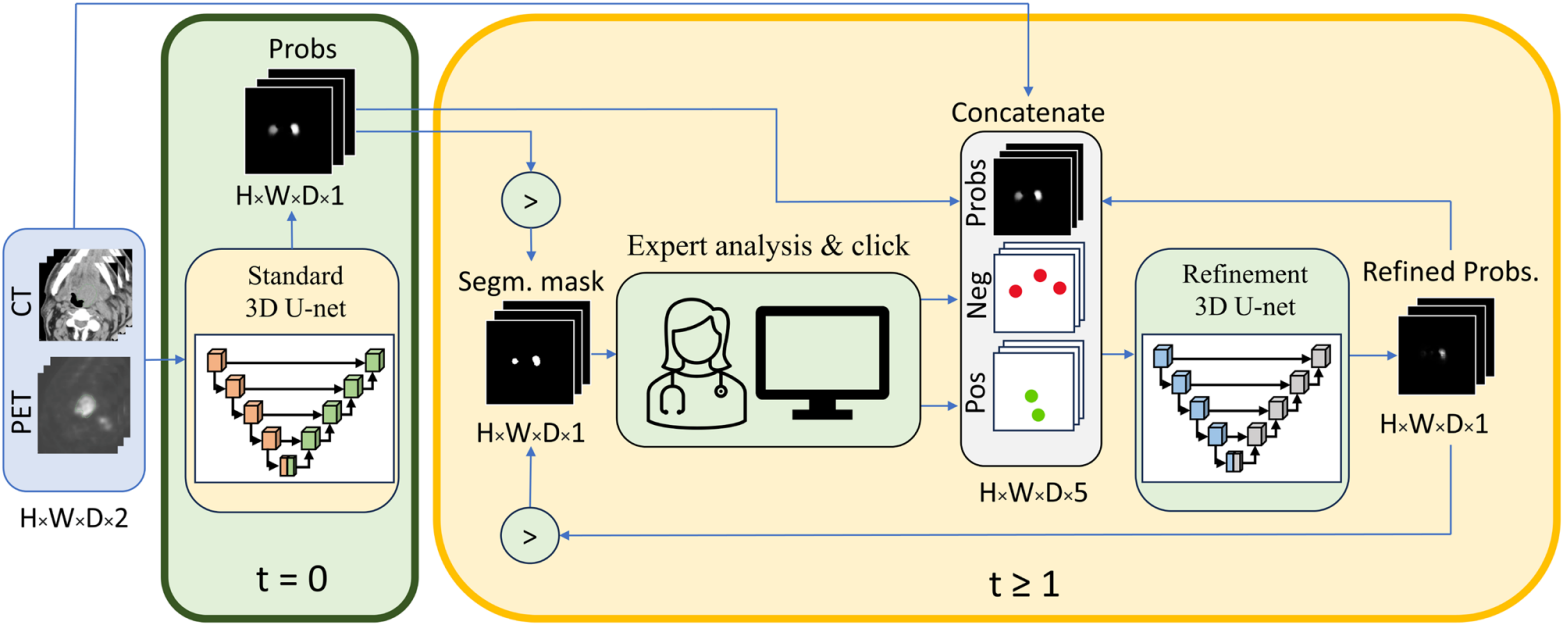
Visualisation of 2S-ICR framework. The initial segmentation ($t=0$) is provided by a standard model which is shown in the green box on left. The segmentation refinement ($t\ge 1$) loop using a refinement model is visualised in the yellow box on right. Spatial dimensions (H$\times$W$\times$D), thresholding (>), negative (Neg), and positive (Pos) feature maps.

The 2S-ICR dual-model segmentation framework, depicted in Fig. 3 and formalized in Algorithm 1, integrates two deep learning models: a standard segmentation model with a 2-channel input and an interactive refinement model with a 5-channel input. If no interactions are given to the 2S-ICR, it segments the PET-CT image using the standard model. When the user first interacts with the model, the given error coordinate, PET-CT image, and the output of the standard model are given to the interactive model. When the user further interacts with the 2S-ICR, the output of the standard model is replaced with the last output of the 2S-ICR, and the new interaction coordinate is given alongside with the previous ones for the 2S-ICR to further refine its output. We train the standard model and the interactive model separately, so as to closely follow the scenario where the 2S-ICR is applied on top of a pre-trained GTVt segmentation network.

A key feature of 2S-ICR is its use of sigmoid-activated segmentation volumes as a memory mechanism. These volumes, with continuous values in the range 0 to 1, initially bridge the standard and interactive models by preserving the spatial information of initial segmentation. During iterative interactions, they maintain continuity between interaction events, stabilizing refinements per user input. Unlike prior methods, such as DeepGrow^12^ and DeepEdit^13^, which lack memory mechanisms and thus cannot leverage prior segmentation states, 2S-ICR’s continuous segmentation maps enable it to examine and utilise prior segmentation state in interpreting the interaction inputs for enhanced performance.

**Algorithm 1:**
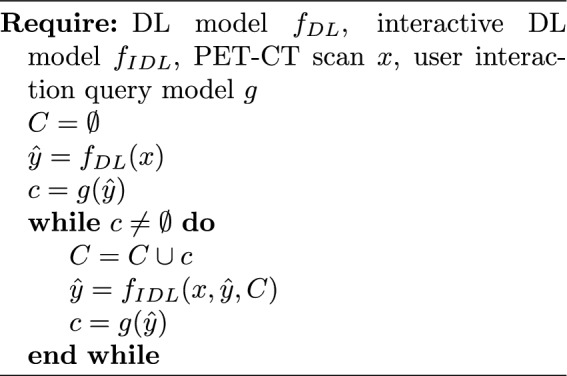
2S-ICR interactive segmentation algorithm

We utilized the Monai implementation of the 3D U-Net architecture^27^ across all models: the initial segmentation model of 2S-ICR framework, the segmentation refinement model of the framework, and the models for DeepGrow and DeepEdit. To ensure a fair comparison between these methods, the only difference was the number of input channels. The networks consisted of channels [16, 32, 64, 128, 256], stride [1, 2, 2, 2], two residual units. The choice of a stride of 1 for the first layer was pivotal for enhancing the impact of the interaction event. All interactive methods use a click encoding scheme proposed by Maninis et al.^28^ for 2D images, adapted by DeepGrow^12^ for 3D volumes, and adopted by DeepEdit^13^ and 2S-ICR. User clicks are encoded as Gaussian-smoothed balls in two 3D volumes: one for positive clicks to mark foreground (e.g., tumors in PET-CT) and one for negative clicks to mark background, guiding precise segmentation refinement.

### Ensemble of interactive segmentation models

The ensemble experiments consist of a five-member ensemble based on the 5-fold cross-validation, both for the standard and interaction models. The ensemble probability is based on the average of the member probabilities. The ensembling was integrated into the interaction loop by selecting each interaction coordinate based on the ensemble prediction, i.e., each member in interaction model is given the same interaction coordinates in each iteration.

### Simulated interactions

As large-scale training and validation of an interactive DL model is not feasible with human interactions, we chose to simulate interactions in these phases. We used the user click interaction simulator proposed in^12,13^. The click simulator compares the model output to the ground truth segmentation in order to select optimal interaction coordinates. Specifically, the simulator first extracts erroneous regions by examining where in the volume the model output and the ground truth differ. Then for each erroneous voxel, the distance to the border of the erroneous region is computed. After this, the distances are normalized by the sum of all the distances. As a result, all voxel values are in the range [0, 1] and sum to 1. Then, we treat these values as probabilities of a multinomial distribution and sample an interaction coordinate accordingly.

### Training procedure

The 2S-ICR framework comprises an initial segmentation network and a refinement network, designed to iteratively enhance segmentation accuracy based on user feedback. To prevent the refinement network from becoming overly reliant on the initial segmentation, we introduce a novel regularization strategy during training. This dependency on the initial mask can hinder the network’s ability to effectively incorporate interaction feedback. Our approach mitigates this issue by randomly omitting the initial segmentation with a probability of $p_{\text {drop}}$, replacing it with a neutral volume filled with the value 0.5. Since the initial segmentation is a post-sigmoid output where each voxel represents the probability of belonging to the foreground class, the value 0.5 signifies uncertainty. This regularization encourages the network to rely more on the original input and user interactions, improving performance and responsiveness to feedback.

We evaluated the impact of varying $p_{\text {drop}}$ on segmentation performance and the number of modified voxels per interaction (Table 5). The results indicate that $p_{\text {drop}} > 0$ consistently yields better outcomes than $p_{\text {drop}} = 0$. The average DSC improved with non-zero values of $p_{\text {drop}}$, and the number and standard deviation of changed voxels per interaction increased, reflecting greater adaptability in the refinement process. Based on these findings, we chose $p_{\text {drop}} = 0.2$ for our experiments.

Unlike previous interactive volumetric segmentation methods^13,29^ that execute the backward pass only after all interaction events have been accumulated–making the process computationally costly–we optimized the refinement network of the 2S-ICR framework at every interaction event during training. This approach significantly accelerated the training process while still allowing the model to achieve state-of-the-art performance.

The initial network of the 2S-ICR is trained without any user interactions and its output segmentation serves as the starting point for subsequent refinements by the refinement network. For the training of the refinement network we follow the procedure proposed in prior research^13^. Specifically, we randomly determine the number of simulated interactions in each training iteration, employing a uniform distribution ranging from 1 to 15 interactions. This approach ensures that the refinement network learns to effectively incorporate user interactions across a diverse range of scenarios. During training, the best checkpoint was chosen based on the highest mean DSC over interactions ranging from 0 to 10, reflecting our primary evaluation metrics as presented in Tables 1 and 2.

All models were trained using a composite loss function that integrates the Dice Loss with the Binary Cross-Entropy (BCE) Loss, similar to previous approaches for OPC GTVt segmentation^7,8^. The composite loss function is formulated as:

1 $$\begin{aligned} \mathcal {L}_{\text {DiceBCE}} = \mathcal {L}_{\text {Dice}} + \mathcal {L}_{\text {BCE}}, \end{aligned}$$

where $\mathcal {L}_{\text {Dice}}$ denotes the Dice Loss and $\mathcal {L}_{\text {BCE}}$ the Binary Cross-Entropy Loss. While the composite loss can also be computed as a weighted sum of its components, we chose to use uniform weights.

The Dice Loss is defined as:

2 $$\begin{aligned} \mathcal {L}_{\text {Dice}} = 1 - \frac{2 \sum _{i} p_{i} g_{i} + \epsilon }{\sum _{i} p_{i} + \sum _{i} g_{i} + \epsilon }, \end{aligned}$$

where $p$ and $g$ represent the model output and ground truth segmentation, respectively, and $\epsilon = 1 \times 10^{-5}$ is a smoothing factor to prevent division by zero. The Binary Cross-Entropy Loss is defined as:

3 $$\begin{aligned} \mathcal {L}_{\text {BCE}} = -\sum _{i} \left[ g_{i} \log (p_{i}) + (1 - g_{i}) \log (1 - p_{i}) \right] . \end{aligned}$$

In both loss functions, the summation is over the voxels of the segmentation volume. The composite loss allows for a more comprehensive optimization by addressing both the overlap of the imbalanced foreground class and the per-pixel classification accuracy^30^.

To enhance the models’ robustness to variations in imaging conditions and reduce overfitting, we applied a data augmentation pipeline during model training, adhering to the procedures presented in^8^. All the models are trained under the same settings for fair comparison. Specifically, we apply random affine transformations that include rotations over all axes up to 45 degrees, scaling and shearing within ranges of [-0.1, 0.1], and translation within a range of [-32, 32] voxels, each with a probability of 0.5. In addition, the augmentations include mirroring over all the axes, induced with the same probability of 0.5. For the CT modality, additional intensity augmentations are implemented, which consist of random contrast adjustments, with gamma range of [0.5, 1.5] and applied with probability of 0.25, intensity shifting, with offset of 0.1 and applied with probability of 0.25, random Gaussian noise, with standard deviation of 0.1 and applied with probability of 0.25, and Gaussian smoothing applied with probability of 0.25.

Each model was trained for a maximum of 300 epochs, employing early stopping with a patience of 50 epochs. We utilized the AdamW^31^ optimization algorithm. The initial learning rate was set to $1 \times 10^{-4}$ and gradually decayed to zero by the final epoch using the cosine annealing scheduler. The AdamW weight decay coefficient was set to $1 \times 10^{-5}$. The best checkpoint of each model was determined based on the highest mean DSC over 0 to 10 interactions, evaluated after each epoch on the validation fold during 5-fold cross-validation. Each model was trained using a mini-batch size of 1 on an NVIDIA A100 GPU with 80 GB of memory. Additional validation runs were performed on an NVIDIA RTX 3080 GPU with 10 GB of memory.

### Evaluation measures

We assess the performance of the algorithms using two widely adopted metrics for automated GTVt segmentation: the Dice Similarity Coefficient and the Hausdorff Distance at the 95th percentile^2^. Given the isotropic 1 mm voxel resolution of both the HECKTOR and MDA datasets, Euclidean distances between voxels correspond directly to distances in millimeters.

Before computing the evaluation metrics, the predicted segmentation probabilities are binarized by thresholding at 0.5 to produce the binary segmentation $S$:

4 $$\begin{aligned} S = \{ i \mid p_i > 0.5 \}, \end{aligned}$$

where $p_i$ is the predicted probability at voxel $i$. This binarization is applied consistently for all evaluation metrics to ensure a fair comparison between the predicted and ground truth segmentations.

The DSC measures the volumetric overlap between the ground truth segmentation and the predicted segmentation. Let $G$ be the set of voxels belonging to the ground truth segmentation:

5 $$\begin{aligned} G = \{ i \mid g_i = 1 \}, \end{aligned}$$

where $g_i$ is the ground truth label at voxel $i$, with $g_i = 1$ indicating the foreground class.

The DSC is defined as:

6 $$\begin{aligned} \text {DSC}(G, S) = \frac{2 |G \cap S|}{|G| + |S|}, \end{aligned}$$

where $| \cdot |$ denotes the cardinality of a set, and $G \cap S$ represents the intersection of the two sets. The DSC ranges from 0 (no overlap) to 1 (perfect overlap), providing a measure of how closely the predicted segmentation matches the ground truth.

The HD95 metric quantifies the spatial discrepancy between the surfaces of the ground truth and predicted segmentations on a per-volume basis. It is a robust version of the Hausdorff Distance, focusing on the 95th percentile of the distances to reduce the impact of outliers.

For each volume $v$, we first extract the surfaces (boundary voxels) of the ground truth segmentation $\partial G_v$ and the predicted segmentation $\partial S_v$ from their respective binary segmentations $G_v$ and $S_v$.

We define the minimum distance from a point to a surface as:

7 $$\begin{aligned} d(a, \partial B) = \min _{b \in \partial B} \Vert a - b \Vert , \end{aligned}$$

where $a$ is a point on one surface, $\partial B$ is the set of points on the other surface, and $\Vert a - b \Vert$ is the Euclidean distance between points $a$ and $b$.

The sets of distances are then:

8 $$\begin{aligned} D_{G_v \rightarrow S_v}&= \{ d(x, \partial S_v) \mid x \in \partial G_v \}, \end{aligned}$$

9 $$\begin{aligned} D_{S_v \rightarrow G_v}&= \{ d(y, \partial G_v) \mid y \in \partial S_v \}. \end{aligned}$$

We combine these distances into a single set for each volume:

10 $$\begin{aligned} D_v = D_{G_v \rightarrow S_v} \cup D_{S_v \rightarrow G_v}. \end{aligned}$$

The HD95 metric for volume $v$ is then defined as the 95th percentile of the distances in $D_v$:

11 $$\begin{aligned} \text {HD95}_v = \operatorname {Percentile}_{95}(D_v), \end{aligned}$$

where $\operatorname {Percentile}_{95}(D_v)$ denotes the value below which 95% of the distances in $D_v$ fall. Since the voxel spacing is isotropic 1 mm, these distances are measured in millimeters.

After computing the HD95 metric for each volume, we aggregate the results by reporting the median and interquartile range (IQR) of the image-level HD95 values:

12 $$\begin{aligned} \text {Median HD95}&= \operatorname {Median} \left( \{ \text {HD95}_v \} \right) , \end{aligned}$$

13 $$\begin{aligned} \text {IQR HD95}&= Q_3 - Q_1, \end{aligned}$$

where $Q_1$ and $Q_3$ are the 25th and 75th percentiles of the set $\{ \text {HD95}_v \}$, respectively.

To evaluate the interaction efficacy of the algorithms, we report the *Number of Clicks* (NoC) required to achieve predefined performance thresholds for both the DSC and HD95 metrics. The NoC quantifies the average number of user interactions needed for each sample to exceed these thresholds.

For the DSC metric, we report the NoC required to reach DSC thresholds of 0.75 and 0.85, which indicate the number of clicks needed to achieve a Dice Similarity Coefficient of 0.75 and 0.85, respectively. Similarly, for the HD95 metric, we report the NoC required to reduce the HD95 below thresholds of 5.0 mm and 2.5 mm, corresponding to the number of clicks needed to bring the Hausdorff Distance at the 95th percentile below 5.0 mm and 2.5 mm, respectively.

The maximum number of allowed clicks is set to 20. If the model fails to reach the target threshold within this limit, the sample is considered a failure. We report the *Percent of Failures* as the percentage of failed samples for each threshold, calculated as:

14 $$\begin{aligned} \text {PoF} = \left( \frac{\text {Number of Failed Samples}}{\text {Total Number of Samples}} \right) \times 100\%. \end{aligned}$$

Specifically, the PoF at DSC thresholds of 0.75 and 0.85 denotes the percentage of samples that did not achieve the respective DSC thresholds within 20 clicks. Likewise, the PoF at HD95 thresholds of 5.0 mm and 2.5 mm represents the percentage of samples that did not reduce the HD95 below the respective thresholds within 20 clicks.

### Datasets

This study does not involve human subjects as it relies on retrospective and registry-based data; therefore, it is not subject to IRB approval. Our external validation dataset was retrospectively collected under a HIPAA-compliant protocol approved by the MD Anderson Institutional Review Board (RCR03-0800), which includes a waiver of informed consent.

The 2021 HEad and neCK TumOR dataset (HECKTOR), introduced in^2^, consists of co-registered PET-CT images from 224 patients. The dataset was gathered from five centers located in Canada, Switzerland, or, France, with ground truth GTVt segmentations provided by multiple annotator agreement. The external MD Anderson Cancer Center dataset (MDA) consists of co-registered PET-CT images from 67 patients that are human papillomavirus positive, with the GTVt segmentations from a single annotator. The images are cropped to contain only the head and neck region, centered on the GTVt, and resampled to $144^3$ volumes with isotropic 1 mm resolution, i.e., in terms of both pixel-spacing and slice thickness.

We adhered to the data normalization procedure established in the previous work^7^. The CT scans were windowed to [-200, 200] Hounsfield units and subsequently normalized to the range of [-1, 1]. The PET scans were standardized using z-score normalization. This normalization procedure ensured consistency across the datasets and enabled the use of the same models without retraining.

## Supplementary Information

Supplementary Information.

## Data Availability

The HECKTOR 2021 training dataset is publicly accessible from https://www.aicrowd.com/challenges/miccai-2021-hecktor. The external validation dataset is publicly available on https://doi.org/10.6084/m9.figshare.22718008.

## Code availability

The 2S-ICR framework and trained models are available on GitLab (https://version.aalto.fi/gitlab/saukkom3/interactive-segmentation).

## References

[1] Nuñez-Vera, V., Garcia-Perla-Garcia, A., Gonzalez-Cardero, E., Esteban, F. & Infante-Cossio, P. Impact of treatment on quality of life in oropharyngeal cancer survivors: A 3-year prospective study. *Cancers* **16**(15), 2724 (2024). 39123452 10.3390/cancers16152724 PMC11311390

[2] Andrearczyk, V. et al. Overview of the hecktor challenge at miccai 2021: aAutomatic head and neck tumor segmentation and outcome prediction in pet/ct images. In *3D Head and Neck Tumor Segmentation in PET/CT Challenge* (ed. Andrearczyk, V.) 1–37 (Springer, 2021). 10.1007/978-3-031-27420-6_1 PMC10171217 37195050

[3] Rasch, C., Steenbakkers, R. & Van Herk, M. Target definition in prostate, head, and neck. *Semin. Radiat. Oncol.* **15**, 136–145 (2005). 15983939 10.1016/j.semradonc.2005.01.005

[4] Cardenas, C. E. et al. Comprehensive quantitative evaluation of variability in magnetic resonance-guided delineation of oropharyngeal gross tumor volumes and high-risk clinical target volumes: an r-ideal stage 0 prospective study. *Int. J. Radiat. Oncol. Biol. Phys.* **113**(2), 426–436 (2022). 35124134 10.1016/j.ijrobp.2022.01.050 PMC9119288

[5] Lin, D. et al. E pluribus unum: prospective acceptability benchmarking from the contouring collaborative for consensus in radiation oncology crowdsourced initiative for multiobserver segmentation. *J. Med. Imaging* **10**(S1), S11903–S11903 (2023). 10.1117/1.JMI.10.S1.S11903 PMC9907021 36761036

[6] Iantsen, A., Visvikis, D. & Hatt, M. *Squeeze-and-Excitation Normalization for Automated Delineation of Head and Neck Primary Tumors in Combined PET and CT Images* 37–43 (Springer International Publishing, 2021).

[7] Sahlsten, J. et al. Application of simultaneous uncertainty quantification and segmentation for oropharyngeal cancer use-case with bayesian deep learning. *Commun. Med.* **4**(1), 110 (2024). 38851837 10.1038/s43856-024-00528-5 PMC11162474

[8] Myronenko, A., Siddiquee, M. M. R., Yang, D., He, Y., & Xu, D. Automated head and neck tumor segmentation from 3d pet/ct. (2022).

[9] Marinov, Z., Jager, P. F., Egger, J., Kleesiek, J. & Stiefelhagen, R. Deep interactive segmentation of medical images: A systematic review and taxonomy. *IEEE Trans. Pattern Anal. Mach. Intell.* **46**, 10998–11018 (2024). 39213271 10.1109/TPAMI.2024.3452629

[10] Wang, R. et al. Medical image segmentation using deep learning: A survey. *IET Image Processing* **16**(5), 1243–1267 (2022).

[11] Wei, Z., Ren, J., Korreman, S. S. & Nijkamp, J. Towards interactive deep-learning for tumour segmentation in head and neck cancer radiotherapy. *Phys. Imaging Radiat. Oncol.* **25**, 100408 (2023). 36655215 10.1016/j.phro.2022.12.005 PMC9841279

[12] Sakinis, T., Milletari, F., Roth, H., Korfiatis, P., Kostandy, P., Philbrick, K., Akkus, Z., Xu, Z., Xu, D., & Erickson, B. J. Interactive segmentation of medical images through fully convolutional neural networks. *arXiv preprint* arXiv:1903.08205 (2019).

[13] Diaz-Pinto, A. et al. Deepedit: Deep editable learning for interactive segmentation of 3d medical images. In *Data Augmentation, Labelling, and Imperfections* (eds Nguyen, H. V. et al.) 11–21 (Springer Nature Switzerland, 2022).

[14] M. J. Cardoso, W. Li, R. Brown, N. Ma, E. Kerfoot, Y. Wang, B. Murrey, A. Myronenko, C. Zhao, D. Yang, V. Nath, Y. He, Z. Xu, A. Hatamizadeh, A. Myronenko, W. Zhu, Y. Liu, M. Zheng, Y. Tang, I. Yang, M. Zephyr, B. Hashemian, S. Alle, M. Z. Darestani, C. Budd, M. Modat, T. Vercauteren, G. Wang, Y. Li, Y. Hu, Y. Fu, B. Gorman, H. Johnson, B. Genereaux, B. S. Erdal, V. Gupta, A. Diaz-Pinto, A. Dourson, L. Maier-Hein, P. F. Jaeger, M. Baumgartner, J. Kalpathy-Cramer, M. Flores, J. Kirby, L. A. D. Cooper, H. R. Roth, D. Xu, D. Bericat, R. Floca, S. K. Zhou, H. Shuaib, K. Farahani, K. H. Maier-Hein, S. Aylward, P. Dogra, S. Ourselin, and A. Feng, Monai: An open-source framework for deep learning in healthcare (2022).

[15] Kerfoot, E. et al. Left-ventricle quantification using residual u-net. In *Statistical Atlases and Computational Models of the Heart. Atrial Segmentation and LV Quantification Challenges* (eds Pop, M. et al.) 371–380 (Springer International Publishing, 2019).

[16] Liu, Q., Xu, Z., Bertasius, G., & Niethammer, M. Simpleclick: Interactive image segmentation with simple vision transformers. In *Proc. IEEE/CVF International Conference on Computer Vision*, pp. 22290–22300 (2023). 10.1109/iccv51070.2023.02037 PMC11378330 39247160

[17] Sun, S., Xian, M., Xu, F., Capriotti, L. & Yao, T. Cfr-icl: Cascade-forward refinement with iterative click loss for interactive image segmentation. *Proc. AAAI Conf. Artif. Intell.* **38**, 5017–5024 (2024).

[18] Sofiiuk, K., Petrov, I. A., & Konushin, A. Reviving iterative training with mask guidance for interactive segmentation. In *2022 IEEE International Conference on Image Processing (ICIP)*, pp. 3141–3145 (2022).

[19] Luan, S. et al. Deep learning for head and neck semi-supervised semantic segmentation. *Phys. Med. Biol.* **69**(5), 055008 (2024). 10.1088/1361-6560/ad25c2 38306968

[20] Andrearczyk, V., Oreiller, V., Hatt, M., & Depeursinge, A. *Head and Neck Tumor Segmentation and Outcome Prediction: Third Challenge, HECKTOR 2022, Held in Conjunction with MICCAI 2022, Singapore, September 22, 2022, Proceedings*, vol. 13626. Springer Nature (2023). 10.1007/978-3-031-27420-6_1 PMC10171217 37195050

[21] Chen, X., Zhao, Z., Zhang, Y., Duan, M., Qi, D., & Zhao, H. Focalclick: Towards practical interactive image segmentation. In *Proc. IEEE/CVF Conference on Computer Vision and Pattern Recognition*, pp. 1300–1309 (2022).

[22] Maier-Hein, L. et al. Why rankings of biomedical image analysis competitions should be interpreted with care. *Nat. Commun.* **9**(1), 5217 (2018). 30523263 10.1038/s41467-018-07619-7 PMC6284017

[23] Selles, M., van Osch, J. A., Maas, M., Boomsma, M. F. & Wellenberg, R. H. Advances in metal artifact reduction in ct images: A review of traditional and novel metal artifact reduction techniques. *Eur. J. Radiol.* **170**, 111276 (2024). 38142571 10.1016/j.ejrad.2023.111276

[24] Han, W., Guo, D., Chen, X., Lyu, P., Jin, Y., & Shen, J. Reducing ct metal artifacts by learning latent space alignment with gemstone spectral imaging data. *arXiv preprint* arXiv:2503.21259 (2025).

[25] Nath, V., Yang, D., Landman, B. A., Xu, D. & Roth, H. R. Diminishing uncertainty within the training pool: Active learning for medical image segmentation. *IEEE Trans. Med. Imaging* **40**(10), 2534–2547 (2021). 33373298 10.1109/TMI.2020.3048055

[26] Wang, H., Chen, J., Zhang, S., He, Y., Xu, J., Wu, M., He, J., Liao, W., & Luo, X. Dual-reference source-free active domain adaptation for nasopharyngeal carcinoma tumor segmentation across multiple hospitals. *IEEE Trans. Med. Imaging* (2024). 10.1109/TMI.2024.3412923 38861437

[27] Çiçek, Ö., Abdulkadir, A., Lienkamp, S. S., Brox, T. & Ronneberger, O. 3d u-net: Learning dense volumetric segmentation from sparse annotation. In *Medical Image Computing and Computer-Assisted Intervention - MICCAI 2016* (eds Ourselin, S. et al.) 424–432 (Springer International Publishing, 2016).

[28] Maninis, K.-K., Caelles, S., Pont-Tuset, J., & Van Gool, L. Deep extreme cut: From extreme points to object segmentation. In *Proc. IEEE Conference on Computer Vision and Pattern Recognition (CVPR)* (2018).

[29] Wang, G. et al. Deepigeos: A deep interactive geodesic framework for medical image segmentation. *IEEE Trans. Pattern Anal. Mach. Intell.* **41**(7), 1559–1572 (2018). 29993532 10.1109/TPAMI.2018.2840695 PMC6594450

[30] Asgari Taghanaki, S., Abhishek, K., Cohen, J. P., Cohen-Adad, J. & Hamarneh, G. Deep semantic segmentation of natural and medical images: A review. *Artif. Intell. Rev.* **54**, 137–178 (2021).

[31] Loshchilov, I. & Hutter, F. Decoupled weight decay regularization. In *7th International Conference on Learning Representations, (ICLR)* (2019).

