# Supplementary material for: Interactive 3D segmentation for primary gross tumor volume in oropharyngeal cancer
Source: Sci Rep. 2025 Aug 5;15:28589. doi: 10.1038/s41598-025-13601-3 (PMC12325674; doi:10.1038/s41598-025-13601-3)
Supplement: Supplementary file 1 — Supplementary Information. [file 41598_2025_13601_MOESM1_ESM.pdf]

Interactive 3D Segmentation for Primary Gross Tumor Volume in Oropharyngeal Cancer

Mikko Saukkoriipi[1], Jaakko Sahlsten[1], Joel Jaskari[1], Lotta Orsmaa[2], Jari Kangas[2], Nastaran Rasouli[2], Roope Raisamo[2], Jussi Hirvonen[3], Helena Mehtonen[3], Jorma Järnstedt[3], Antti Mäkitie[4], Mohamed Naser[5], Clifton Fuller[5], Benjamin Kann[6,7], and Kimmo Kaski[1,8*]

[1]Department of Computer Science, Aalto University School of Science, Espoo, Finland

[2]Faculty of Information Technology and Communication Sciences, Computing Sciences, University of Tampere, Tampere, Finland

[3]Department of Radiology, Tampere University, Faculty of Medicine and Health Technology, Tampere University Hospital, Tampere, Finland

[4]Department of Otorhinolaryngology–Head and Neck Surgery, Research Program in Systems Oncology, Faculty of Medicine, University of Helsinki and Helsinki University Hospital, Helsinki, Finland

[5]Department of Radiation Oncology, The University of Texas MD Anderson Cancer Center, Houston, TX USA

[6]Artificial Intelligence in Medicine Program, Mass General Brigham, Harvard Medical School, Boston, MA, USA

[7]Department of Radiation Oncology, Dana-Farber Cancer Institute and Brigham and Women's Hospital, Harvard Medical School, Boston, MA, USA

[8]The Alan Turing Institute, British Library, 96 Euston Rd, London NW1 2DB, United Kingdom

[*]Corresponding author

July 24, 2025

Supplementary Results

Even though all methods were trained with a maximum of 15 interactions, we can see that results continue to improve beyond this point. The 2S-ICR method demonstrates the strongest Dice performance across all 0 to 20 interactions, as shown in the line plots above and in Figures 1 and 2.

In Figure 3, improvements in segmentation are observed across all instances after interaction. Noteworthy enhancements are particularly evident in samples with initially suboptimal segmentations. Importantly, all samples demonstrated either consistent or improved segmentation accuracy, with no cases exhibiting a decline in quality due to the interaction process.

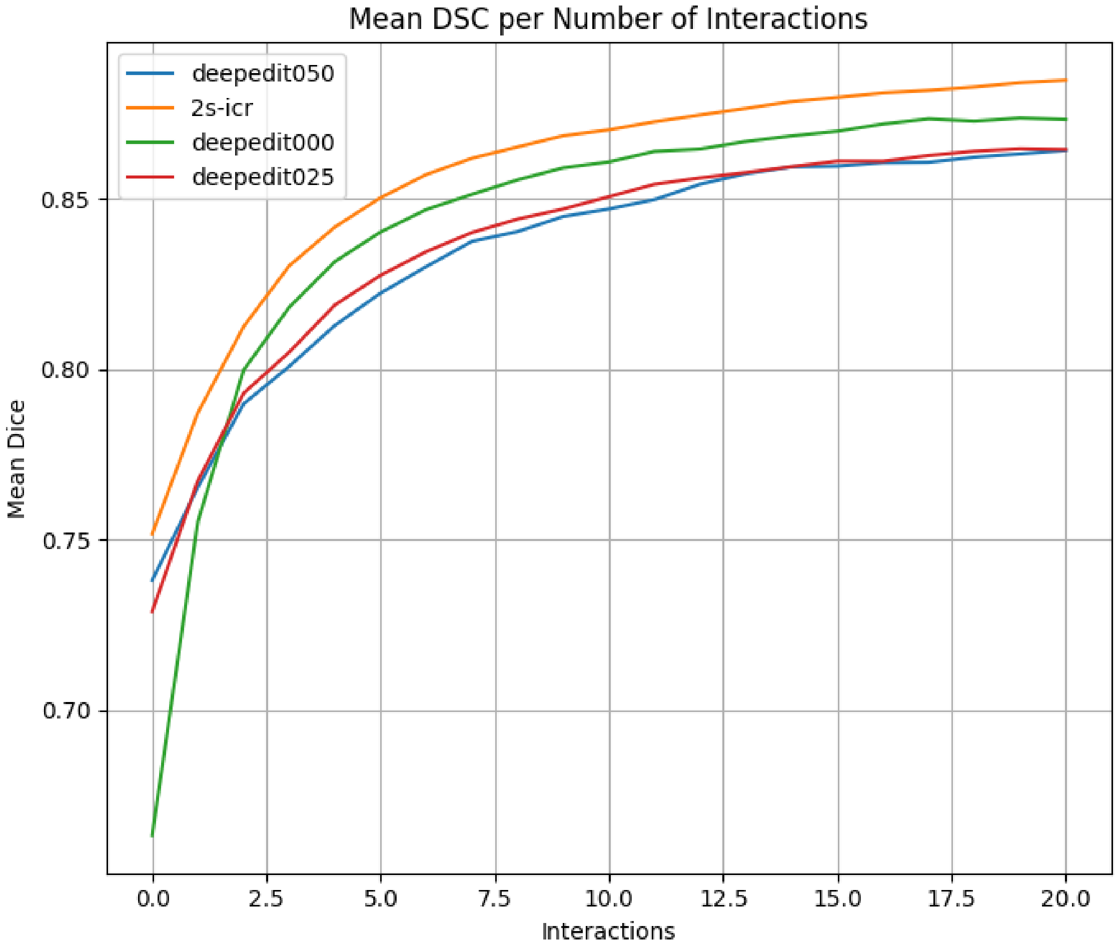

Figure 1: Mean Dice coefficient across 0 to 20 interactions on the Hecktor 2021 dataset (N = 224).

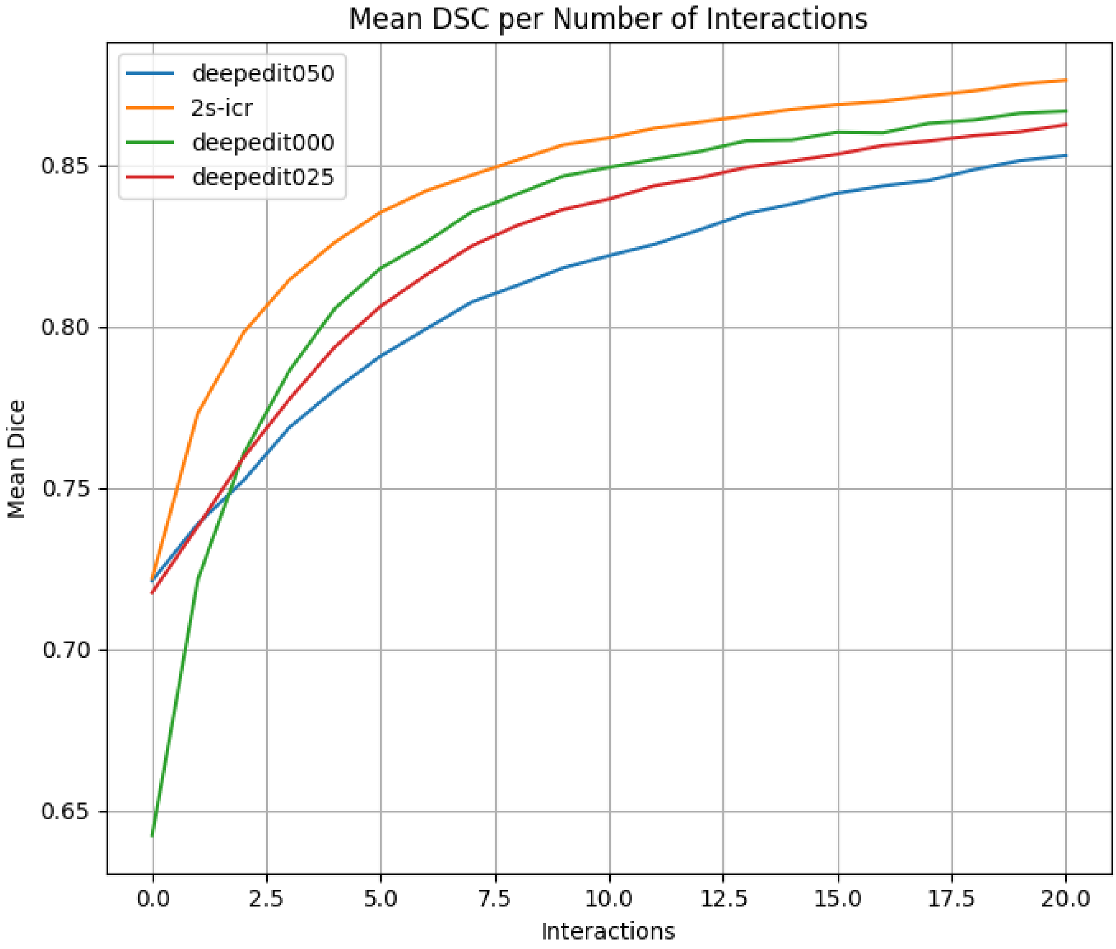

Figure 2: Mean Dice coefficient across 0 to 20 interactions on the MDA dataset (N = 67).

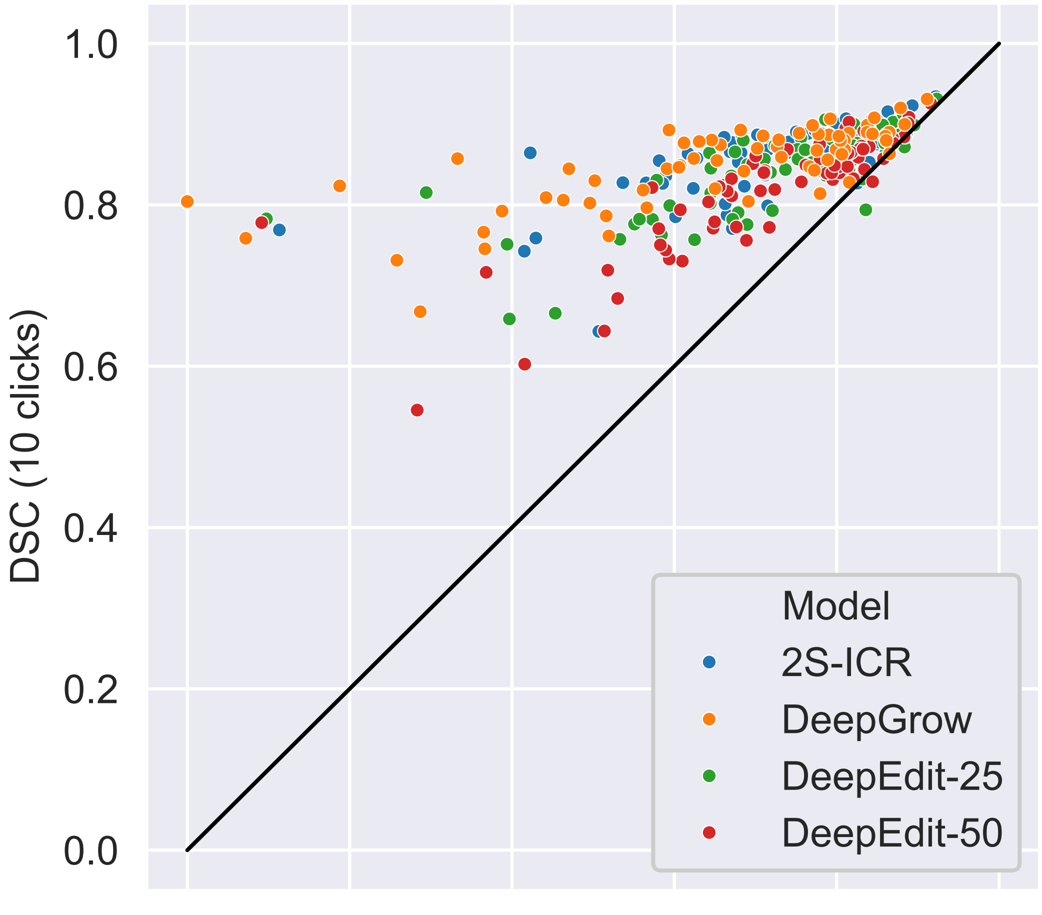

Figure 3: Change in Dice similarity coefficient (DSC) on individual samples from the MDA dataset.
